# When COVID-19 came to town: Measuring the impact of the coronavirus pandemic on footfall on six high streets in England

**DOI:** 10.1177/23998083211048497

**Published:** 2022-03

**Authors:** Marcus Enoch, Fredrik Monsuur, Garyfalia Palaiologou, Mohammed A Quddus, Fiona Ellis-Chadwick, Craig Morton, Rod Rayner

**Affiliations:** 5156 Loughborough University, Loughborough, Leicestershire, UK; Proximity Futures, Hinckley, Leicestershire, UK

**Keywords:** High street vitality, town centre retail decline, coronavirus outbreak, COVID-19 pandemic, Wi-fi footfall monitoring sensors, Prais–Winsten AR(1) dynamic time series model

## Abstract

Town centres in the economically developed world have struggled in recent years to attract sufficient visitors to remain economically sustainable. However, decline has not been uniform, and there is considerable variation in how different town centres have coped with these challenges. The arrival of the coronavirus (COVID-19) pandemic public health emergency in early 2020 has provided an additional reason for people to avoid urban centres for a sustained period. This paper investigates the impact of coronavirus on footfall in six town centres in England that exhibit different characteristics. It presents individual time series intervention model results based on data collected from Wi-fi footfall monitoring equipment and secondary sources over a 2-year period to understand the significance of the pandemic on different types of town centre environment. The data show that footfall levels fell by 57%–75% as a result of the lockdown applied in March 2020 and have subsequently recovered at different rates as the restrictions have been lifted. The results indicate that the smaller centres modelled have tended to be less impacted by the pandemic, with one possible explanation being that they are much less dependent on serving longer-distance commuters and on visitors making much more discretionary trips from further afield. It also suggests that recovery might take longer than previously thought. Overall, this is the first paper to study the interplay between footfall and resilience (as opposed to vitality) within the town centre context and to provide detailed observations on the impact of the first wave of coronavirus on town centres’ activity.

## Introduction

Town centres around the world have struggled in recent years to attract sufficient visitors to remain economically sustainable. Reasons for this include a legacy of over expansion before 2010; higher fixed operating costs: wages, rents and business rates; market uncertainties and economic recession; and changing consumer tastes and changing shopping habits, evidenced by the growth in the number of out-of-town outlets and more recently a rise in online spending (Turner and Corstorphine, 2020). Thus, fluctuations in visitor numbers (footfall) have significantly affected UK high streets. Hence, July 2019 experienced a reduction in footfall of 1.9%, the worst July performance for 7 years, whilst the vacancy rate of premises in the same month was 10.3%, its highest level since January 2015 (BBC News, 2019), figures likely influenced by the net decline of 1234 chain stores on Britain’s top 500 high streets during the previous 6-month period – the highest number of closures since 2010 (PWC, 2019). In short, there are too many (large) shops that are situated in the wrong locations. However, such decline has not been uniform and there is considerable variation in how different centres have coped with these challenges (Tselios et al., 2018). Meanwhile, the arrival of the coronavirus or COVID-19 pandemic public health emergency in early 2020 has provided an additional reason for people not to congregate in urban centres for a sustained period. This is reflected by the fact that by July 2020, footfall had fallen by close to 50% of the 2019 figure (ONS, 2020) dealing a potentially devastating blow to UK high streets.

Details of how the early phases of the COVID-19 pandemic crisis unfolded in England are provided in Table 1. From this, perhaps the two major events were the enforcement of the lockdown on 23 March 2020 and the re-opening of most non-essential shops on 15 June 2020.

**Table 1.** Key coronavirus pandemic events in England: 31 January 2020–1 August 2020.

Key dates	Key events in England
31/01/2020	***First cases of COVID-19 confirmed***
05/02/2020	*First transmission of COVID-19 announced*
05/03/2020	*First COVID-19 death announced*
15/03/2020	*Over 70s asked to self-isolate*
16/03/2020	*PM Boris Johnson encourages social distancing*
	*People urged to stop social interaction, non-essential travel, and work at home if possible*
	*People to avoid pubs, clubs and theatres*
	*Vulnerable groups must self-isolate*
20/03/2020	*Lockdown measures introduced*
	*Furlough scheme introduced*
	*Bars, pubs, restaurants, cinemas, gyms and nightclubs close*
23/03/2020	***Lockdown enforced***
	*No 2+ people gatherings allowed*
	*People only to leave home to shop for basic necessities, travel to essential jobs or exercise (max 1 h)*
	*Shops selling non-essential goods close*
25/03/2020	*Coronavirus Act passed. Police given powers to enforce and interfere with individual rights and liberty*
26/03/2020	*New police powers come into effect*
	*Restricted selling of non-essential goods allowed*
31/03/2020	*Police issue guidelines to encourage public to comply with lockdown*
05/04/2020	*Queen delivers address to the nation: ‘We must remain united and resolute’*
12/04/2020	*6428 attributed deaths in England – the highest weekly total of the pandemic*
16/04/2020	*Lockdown measures extended for 3 weeks*
21/04/2020	*England passes 20,000 attributable deaths*
22/04/2020	No longer illegal to leave home without a reasonable excuse
	People may exercise more than once a day and visit parks
10/05/2020	PM Boris Johnson announces roadmap for re-starting the economy
	Shops allowed to re-open with social distancing in place
11/05/2020	People urged to return to work if they cannot work from home
	Unlimited exercise and some sports allowed
	Garden centres open
	More sectors of the economy returning to work
01/06/2020	People allowed to meet with 6 others from separate households
	Schools re-open for Years 1 and 6
	Car showrooms and outdoor markets open
05/06/2020	*England passes 40,000 attributable deaths*
03/06/2020	Grandparents who live alone can mix with their family
	Non-cohabiting couples can stay together overnight
15/06/2020	*Non-essential shops re-open*
	*Face coverings compulsory on public transport*
19/06/2020	Alert level reduced from 4 to 3
24/06/2020	PM Boris Johnson announces list of business allowed to re-open on 4th Jul
	Social distance rule relaxed from 2 to 1+ metres
29/06/2020	*First local lockdown imposed in Leicester*
04/07/2020	Pubs, cafes, bars and personal care businesses re-open
06/07/2020	People who are shielding can meet up to 6 people outside and create a support bubble
25/07/2020	*Face coverings compulsory in all enclosed public spaces and shops*
31/07/2020	*Regional lockdown applied in Greater Manchester and West Yorkshire*
01/08/2020	Shielding ends. Vulnerable people can leave their homes

While town centre performance is a complex and multi-dimensional concept, recent research suggests that footfall measurements enable a dynamic understanding of town centre activity and offer a reliable proxy for assessing performance (Mumford et al., 2020). In a similar vein, this paper considers footfall as a core indicator for town centre activity and by extension urban resilience. Accordingly, this paper considers footfall activity in six disparate town centres in England and develops comprehensive models for analysing footfall as a multi-dimensional construct. The aim is to explore the potential for footfall to reveal insights on the differential response and vulnerability of high streets to changes triggered by the pandemic shock and to help inform a roadmap to their recovery (Ntounis et al., 2020). It is a first step towards creating a footfall-based town centre resilience measurement model.

## The interplay between town centre footfall and resilience

As town centres in many countries around the world begin to switch between sustained periods of lockdown and periods of near-normal operation, the impact of the COVID-19 pandemic on trade starts to become apparent. Economic indicators suggest the UK is entering a period of recession, but some retail businesses have benefited during the pandemic. Varying retailing performance indicates that town centres and high streets – typically being retail-focused – have been affected to differential degrees (Stephenson, 2020). Reasons for this include, for example, the agglomerations and types of retail and service provision (Dolega et al., 2019), their e-presence (Doherty and Ellis-Chadwick, 2010; Jones and Livingstone, 2018) or e-resilience (Singleton et al., 2016). Meanwhile, in a study that explored the effects of the 2007/8 economic recession on UK high streets, Tselios et al. (2018) found that the impact on those centres that experienced lower retail vacancy rates in the pre-recession period were more negatively affected but that these effects were moderated by the size and location of the retail centre. In spite of this evidence, the ways in which town centre activity relates to their vulnerability against economic shock, and subsequently to their capacity and trajectory to recovery, remain understudied.

The lockdown imposed by the UK government on 23 March 2020, as a measure to mitigate the spread of COVID-19 is of particular interest to town centre studies because it offers a possibility to investigate timely gaps in knowledge with regards to town centre resilience. Broadly, resilient towns are those which can respond effectively to economic and social change by being flexible and adaptive (CLES and ATCM, 2015) while also developing new paths to achieve growth (Boschma, 2015). The latter point highlights an evolutionary perspective on (regional) resilience ‘that connects shocks to the determinants of the ability of regions to develop new growth paths’ (ibid., p. 743).

The first gap relates to the way town centre resilience is conceptualised and measured. Town centre resilience is currently understood in relation to vitality and viability (Wrigley and Dolega, 2011) and measured primarily through vacancy rates but these are typically difficult to determine and often indicate the viability of the premises in terms of rental charges, condition and location of property rather that giving a true indication of the vitality of business activity (Findlay and Sparks, 2010). Furthermore, the time cycles for vacancy churn vary but regardless are typically measured on a bi-annual or annual frequency (e.g. LDC, 2018, 2019). They are also often reported in the form of generic percentages, thus failing to pick up the necessary temporal and spatial granularity required for nuanced town centre management decision-making and responsive interventions and dynamic placemaking strategies. Second, town centre resilience studies currently lack in-depth understanding of the short-term recovery process of town centres from economic shocks and how it relates to a town centre’s capacity to adapt and formulate new growth paths (Dolega and Celińska-Janowicz, 2015). Observations on the immediate and short-term responses of town centres to the COVID-19 lockdown rules are an essential basis that can inform future preparedness and resilience-building strategies, as well as support the contextual understanding of the subsequent phases of reorientation and development. Third, whereas footfall monitoring has a lot to contribute to dynamic place management (Murcio et al., 2018), it has not been explored as an indicator of town centre vulnerability and/or resilience; rather, it has primarily been used to measure town centre attractiveness or study mobility patterns (Millington et al., 2015; Mumford et al., 2020). A summary of footfall studies in the context of high streets, town centres and public spaces is available in Table A1 in the Appendix. In general, the scope of these studies aligns with the aforementioned vitality-focused assessment of town centre activity. In turn, an examination of footfall data from the lens of resilience might reveal new insights about the ways in which continuous real-time data can support local authorities to proactively manage places; for example, by utilising state-of-the-art modelling and forecasting capabilities (see Kontokosta, 2016; Kontokosta and Johnson, 2017).

Alongside their potential, footfall data and measurements show limitations that relate to the sensor infrastructure. For example, GPS sensors offer spatial precision but can observe only localised activity, whereas Wi-fi probes face challenges in terms of errors, typically from overcounting or undercounting due to reliance on and randomisation of mobile devices’ signal signatures which compromises accuracy and the distinction between new and repeat visitors (Soundararaj et al., 2020). These issues are further discussed in the data collection section. Furthermore, to understand town centres as social and economic structures, town centre activity needs to be studied beyond simply counting visitors. It is necessary to also examine other variables affecting human activity such as daily and seasonal behavioural patterns (e.g. weekdays vs. weekends and bank holidays), the impact of weather, as well as the town centre profile in terms of population structure and retail offer. A gap remains in terms of high-resolution studies that consider visitor count fluctuations against the local nuances of footfall and the ways in which human activity is shaped by the socio-economic context and spatial setting of high streets and town centres. To this end, a high-resolution study of footfall, comparatively for selection of towns which all have their own socio-economic characteristics and spatial function, is lacking.

## COVID-19 and footfall

Mobility within some urban settings is heavily monitored with a suite of interconnected systems capable of tracking movements around cities (Schaick et al., 2008). The data collected from these systems provide a useful lens through which to evaluate how the introduction of lockdowns by governments to limit the spread of COVID-19 altered everyday activity patterns. Preliminary research is starting to be published on this topic which provides context to the study reported in this article.

As would be expected, the imposition of restrictions to limit social interaction in many countries during the pandemic (e.g. curfews and shelter in place orders) coincided with a dramatic fall in traveller circulation, as evidenced by the use of time series charts across the media showcasing the effectiveness of lockdowns in curtailing the movement of citizens (Molloy et al., 2021). For instance, in their study of lockdown conditions in Santander, Spain, found that movements decreased by 76% (Aloi et al., 2020). However, this decrease was not evenly distributed across transport modes, with walking declining from 42% of all conducted journeys pre-lockdown to 19% post-lockdown. A similar overall decline is reported in an examination of urban mobility in Columbia, where movements fell by 80% following government-imposed restrictions (Arellana et al., 2020). The magnitude of the decline did vary by journey purpose, with retail and recreation trips declining the most and recovering the slowest. While broad-brush statistics can provide an overall picture of the impact of activity curtailment, it is possible for movements to be affected to a greater or smaller degree in different spatial contexts. This is revealed in an examination of the impact of the COVID-19 lockdown on population density in Sapporo, Japan through the use of mobile phone signals to triangulate the position of urban residents (Arimura et al., 2020). The findings of their analysis showcase how the concentration of citizens shifted to different areas within the city post-lockdown, with the population density in the central business district declining by 55–65% based on the period of the day.

While preliminary research has illustrated some of the spatial–temporal trends in urban mobility in the period immediately following the imposition of lockdowns (Hadjidemetriou et al., 2020), to date no multivariate analysis has been conducted which isolates the magnitude of the effect from other relevant factors (e.g. weather conditions and seasonality). Thus, the analysis reported in this paper offers a more precise measurement of this impact through the application of time series modelling to disentangle the lockdown effect while also shedding light on how the impact varies across different urban settings.

Overall, this research explores the aforementioned gaps in several ways. First, it examines footfall from a resilience perspective and looks at the shifting narratives of attractiveness and vulnerability in the context of the COVID-19 lockdown as an external shock. Second, it uses state-of-the-art Wi-fi probe tracing with the capability to pick up signals even from devices that are switched off, which significantly extends the coverage of such technologies across the population when compared with other nominally similar systems, and to distinguish between the profile of new and frequent visitors. Third, it develops a time series model to observe human activity by cross examining not only footfall across multiple locations but also other spatio-temporal factors and contextual variables that impact town centre activity. The next section provides a detailed description of the data collection process, the data analysis methods and the reasons for their selection.

## Data

For this study, footfall data were taken from a town centre monitoring platform called GEO-Sense that has been deployed in each location in order to monitor town centre performance (see https://proximityfutures.com/geo-sense/). GEO-Sense works in real time by listening for the wireless handshake emitted by mobile devices to see if there are any wireless networks available to connect to. Each connection is logged along with the unique ID (MAC Address) of a device, date, time and the TAG sensor. In dealing with the randomisation of MAC addresses, GEO-Sense employs proprietorial software that can determine which MAC addresses are randomised and which are not, and is thus able to circumnavigate the randomisation systems in place on Apple, Google and other mobile device manufacturers. Finally, it is important to note that for GEO-Sense to capture the data there is no need for the member of public to connect to or authenticate to any type of wireless network, the sensors simply listen. The range of each sensor is adaptable to its location, and as each sensor has its own TAG ID it is possible to monitor movement between sensors.

In terms of performance, it is true that undercounting can occur where people do not have a device with a MAC address – for example, for children or older people. Some people may also not be counted in less densely covered locations for instance if they only use less trafficked streets, as sensor coverage is usually focused on busier thoroughfares. Meanwhile, overcounting may occur should visitors be carrying two or more devices. Other Wi-fi-enabled systems can also sometimes interfere initially to increase the counts for example, from printers, wireless speakers, handheld scanners and from household devices like laptops or games consoles from people living in residences close to the sensors (especially during lockdown). To address the second issue, GEO-Sense filters all devices based on the start of the MAC address and is trained to be able to ignore the non-personal sources within 24 h of their first appearance. Despite these challenges however, internal research by Proximity Futures suggests that footfall numbers provided by GEO-Sense have an accuracy of 96%. This figure was determined during a validation exercise whereby the footfall counts were compared with data from ∼60,000 ticket sales to a large outdoor tourist attraction in the south of England equipped with barrier-only access over the period of a month in the summer of 2018. With regards to ensuring the privacy of town centre visitors, GEO-Sense captures the MAC address of every device within range which is then immediately masked at the sensor level to anonymise the data and hence meet with GDPR requirements. No personal data are obtained – only a link to each device which enables it to be tracked from one location to the next within a closed system of sensors. There is therefore no need for permission to be obtained from town centre visitors in collecting and using the data. As a further level of security for people’s personal privacy, the records for each 24-hour period for each sensor are then re-masked at 3 a.m. each day to ensure that the initial level of masking can never be reverse engineered.

One last point about the data from the sensors is that the number of sensors used in each town remained constant throughout the time-period analysed, with the footfall counts aggregated in each case and for each town’s model. Beyond that, sample bias due to the different number of sensors in each town was not accounted for.

Regarding variables used, the dependent variable was aggregated daily footfall in each town centre.

Next, several COVID-19-related variables were developed. First, the number of deaths attributed to COVID-19 were sourced from official UK Government statistics (see UK Government, 2020). Second, three dummy variables as defined below:

• COVID-19 confirmed: The time period between the first COVID-19 case confirmed, at the 31 January 2020, and the start of the lockdown, at 23 March 2020.
• Lockdown: The time period covering the national lockdown restrictions, from the 23 March 2020 to the 18 June 2020.
• Re-opening of non-essential shops: The time-period covering the reopening of the shops, from the 15 June 2020 to the end of the data series

Next, weather data (temperature and precipitation) were also captured by the GEO-Sense platform for each town from the Dark Sky weather service (see https://darksky.net/).

Finally, dummy variables were also created to represent the occurrence of temporal events, covering weekends, public holidays and university terms.

In all instances, data were aggregated for each day over a 2-year period (unless otherwise noted in Table 2). One other key assumption that needs to be mentioned is that in reality trip attractiveness characteristics to do with the retail offer, or level of employment in towns for instance, will change over time, whereas here we did not have sufficiently granular data (i.e. weekly or monthly) to capture these effects.

**Table 2.** Town centre/case studies profiles.

Town/city	Region	Population 2011 Census/Catchment within 30-min drive	Median age/% aged over 65	Index of Multiple Deprivation (IMD) 2019*	General profile	Number of sensors/period of data monitoring
Hereford	West Midlands	60,415/194,192	47.4/24.7%	137	Cathedral city in a largely rural area	12/(> 24 months)
Loughborough	East Midlands	59,932/1,633,185	38.4/18.1%	244	University town located between Leicester, Derby and Nottingham	7/(> 24 months)
Norwich	East of England	213,166/417,198	33.5/15.1%	61	Regional centre in a largely rural area	10/(since Feb 2019)
Nuneaton	West Midlands	92,698/1,824,009	41.3/19.3%	101	Industrial town located near Coventry and Birmingham	9/(>24 months)
Stockport	North West	283,275/1,737,965	42.4/20%	154	Metropolitan borough within the Greater Manchester conurbation	10/(since Nov 2018)
Welwyn Garden City	East of England	59,910/1,597,495	35.7/15.5%	215	New town with mixed economy within easy commuting distance of London	5/(since Oct 2018)

Note: * Shows local authority rank (MHCLG, 2019). The lower the IMD number, the more deprived the local authority is (out of 317 local authorities).

## Method

GEO-Sense data currently collects footfall data from more than 2 million unique individuals per week in over 60 locations around the UK, of which 40 locations have had monitoring in place for a significant length of time. In sampling the six towns for the study, a systematic approach was adopted which comprised three stages. First, each of the 40 towns equipped with the footfall sensors was categorised in terms of administrative region (e.g. East of England, North West and East Midlands); population size (e.g. less than 100,000; 100,000–200,000 and > 300,000) and ‘function’ (e.g. University, rural, industrial and commuter). Second, we grouped the towns into loosely defined homogenous groups to provide as broad a range of archetypes as possible from the towns available – noting that these towns are by no means representative of settlements across the country. Third, we selected the town in each group which provided us with the most comprehensive level of data in terms of sensor coverage in each town centre and longevity of uninterrupted data provision. The characteristics of the towns/cities modelled can be seen in Table 2.

Once selected, descriptive statistics were applied to the data and reported. The second step was then to develop a series of time series models in order to explain the factors affecting footfall numbers in town centres in light of the COVID-19 pandemic.

The Prais–Winsten AR(1) model was then applied to the data for each case in turn. This is a dynamic time series model which is capable of resolving first order autoregressive (AR1) correlation in the time series data. This makes the residuals stationary, resulting in more reliable and accurate predictions. The model is estimated by a generalised linear square regression. Once a model is estimated, the Durbin–Watson statistic can be calculated to diagnose whether the residuals are serially correlated. The Durbin–Watson statistic should be between 0 and 4, where 0 indicates perfect negative correlation, 2 represents no autocorrelation and a value towards 4 represents perfect positive autocorrelation.

As noted above, the dependent variable in each of the models represents the observed daily footfall occurring in the high street. The independent variables cover observations of weather conditions (temperature and precipitation), calendar events (weekends, bank holidays and term times) and COVID-19 restrictions. In each instance, a parsimonious model is achieved whereby the independent variables which do not display a significant affect over footfall are removed from the analysis.

The model can be written as

$Y_{t}=\beta_{0}+\sum_{j=1}^{k}\beta_{j}X_{jt}+\mu_{t}$ where $\mu_{t}=\rho \mu_{t-1}+e_{t}$

$Y_{t}$ = Daily footfall in time *t*

$\mu_{t}$ = model error term; where *t* = 1, …, *n*

$X_{jt}$ = Vector of explanatory variables

k = number of explanatory variables

ρ = Autocorrelation coefficient of order 1 (i.e. AR1). ρ = 1 means that there is a perfect correlation among residuals.

$\beta_{0},\;\beta_{j}$ = Parameters to be estimated;

$e_{t}$ = i.i.d. error term with zero means and constant variance.

## Results

**Figure 1.**
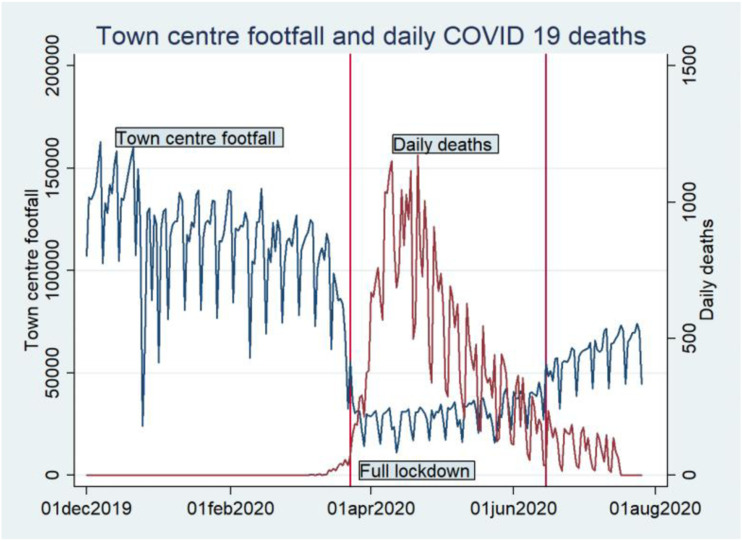
Footfall averages from 1 December 2019 to 1 August 2020 for all six towns/cities which shows the impact of the COVID-19 lockdown.

Figure 1 illustrates how the combined footfall for all six town and city centres was affected by the COVID-19 lockdown.

**Figure 2.**
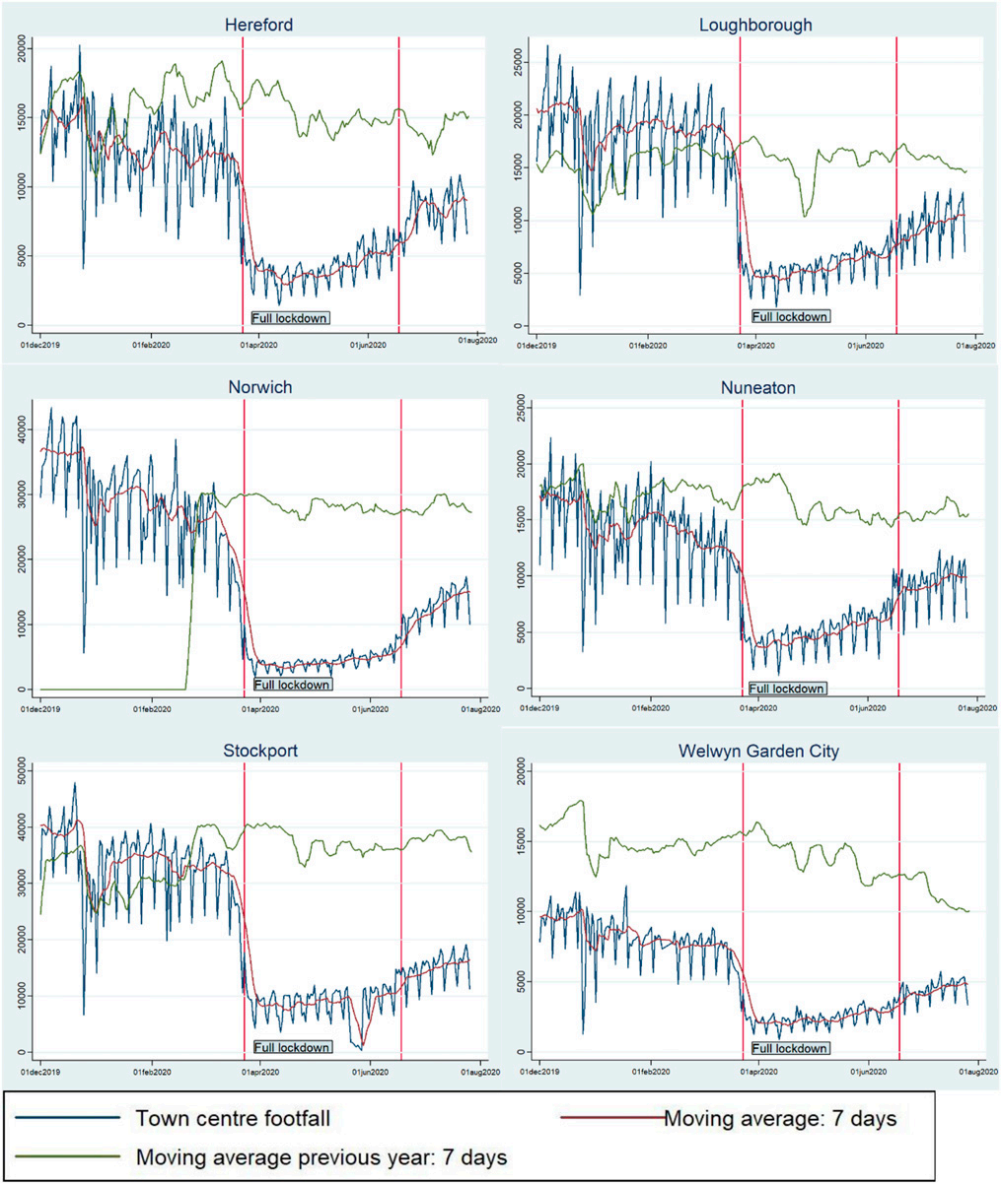
Footfall averages from 1 December 2018 to 1 August 2019; and 1 December 2019 to 1 August 2020 (which shows the impact of the COVID-19 pandemic).

Next, Figure 2 shows how the effect of the COVID-19 lockdown varied across the six cases studied. A 7-day moving average trend line is inserted to smooth out the weekly seasonality inherent in the data. Two separate moving averages are depicted, the first being for 2019 and the second for 2020. Table 3 helps to interpret these figures further.

**Table 3.** Initial estimates of the effect of the COVID-19 lockdown on footfall.

	Mean footfall pre-lockdown (1 Mar 2019–2022 Mar 2020)	Mean footfall since lockdown (23 Mar 2020––27 Jul 2020)	Percentage change (%)	Percentage 7 days’ moving average footfall recovered (27 Jul 2020 compared to 27 Jul 2019) (%)
Hereford	14,401	5540	−61.5	60.5
Loughborough	17,528	6953	−60.3	72.0
Norwich	28,837	7110	−75.3	55.0
Nuneaton	15,250	6506	−57.3	65.0
Stockport	36,178	10,701	−70.4	45.0
Welwyn Garden City	10,638	3116	−70.7	45.6

* For Welwyn Garden City: Debenhams (a large department store) closed its branch in April 2019, and from that moment on footfall fell significantly for that town centre even before the pandemic. This could be why the moving average for 2019 is so much higher compared to 2020.

* For Norwich: The data start in March 2019; hence, the 7 days’ moving average for the previous year is zero before March 2019.

Table 3 shows the mean footfall from 1 March 2019 to the lockdown in March 2020 and then compares this with the mean footfall after 23 March 2020 until 27 July 2020, and shows that the average daily reduction in footfall ranges from around 60% in Hereford, Loughborough and Nuneaton, up to 70–75% in Norwich, Stockport and Welwyn Garden City.

The final column compares the 7 days’ moving average of the last day in the dataset (27 July 2020) and the 7 days’ moving average from the year before (27 July 2019) to get a sense of the recovery of footfall. Here, it would seem that Stockport and Welwyn Garden City are still heavily affected by the restrictions with footfall levels only 45% of those in the same week in the previous year – though one point to note is that Welwyn Garden City was already experiencing a steady drop in footfall prior to COVID-19, perhaps as a result of the closure of a major department store in the town. Next, Norwich (55%) and Hereford (60%) are doing slightly better, whilst the most resilient centres appear to be Nuneaton (65%) and Loughborough (72%) – here, it is necessary to note that, prior to the pandemic, Loughborough seems to have performed much better from October 2019 than from October 2018. These two case studies are the only town centres out of the six examined which are identified in the 2018 Retail Centre Typology classification (Dolega et al., 2019) as ‘mass market and value retail large centres’. The remaining four – Hereford, Norwich, Stockport and Welwyn Garden City – are classified as ‘premium shopping and leisure destinations’. Therefore, these results highlight how utilitarian shopping and convenience took priority over premium shopping and leisure during the pandemic.

Interestingly, there appears to be no obvious link between footfall recovery rate and age profiles nor levels of deprivation (both at the local authority level). However, Welwyn Garden City aside, it does seem that the smaller towns have generally fared better than did Stockport and Norwich. This effect could possibly be due to those larger centres being proportionally more reliant firstly on more discretionary shopping trips and secondly on commuters who might normally travel into larger centres to work (and then shop at lunchtime) – journey purposes which have both been especially strongly affected by the crisis.

Table 4 displays the modelling results for each selected town/city. To ensure consistency across the models, the dependent variable was transformed to a logarithmic scale and the same set of explanatory variables (except the Loughborough model in which a variable – university term time was added) was pursued, regardless of statistical significance (Ziliak and McCloskey, 2008). The model fit is extremely good for all models (with the adjusted *R*^2^ consistently around 0.8, except for the Welwyn Garden City model in which this is 0.66) and the Durbin–Watson statistics indicate that there is almost no autocorrelation present in the models. All the models cover a time-period of 505 days, starting on the 1 March 2019 and ending on the 17 July 2020.

**Table 4.** Model outputs of the effect of the COVID-19 lockdown on footfall.

Dependent variable	Hereford ln (town centre footfall	Loughborough ln (town centre footfall	Norwich ln (town centre footfall	Nuneaton ln (town centre footfall)	Stockport ln (town centre footfall)	Welwyn Garden City ln (town centre footfall)
Precipitation	−0.002	−0.004**	−0.00003	−0.004**	−0.001	−0.001
University term time	—	0.143**	—	—	—	—
Saturday	−0.009	0.099**	−0.005	0.138**	−0.082**	0.184**
Sunday	−0.418**	−0.428**	−0.481**	−0.567**	−0.408**	—
UK COVID-19 case confirmed	−0.200**	−0.055	−0.272**	−0.183**	−0.229*	−0.413**
Lockdown	−0.990**	−0.925**	−1.434**	−0.898**	−1.04**	−1.224**
Non-essential shops re-open	−0.406**	−0.352**	−0.530**	−0.328*	−0.371**	−0.585**
Daily COVID-19 deaths	−0.0004**	−0.0003**	−0.0005**	−0.0004**	-0.0003*	−0.0002
Constant term	9.628**	9.702**	10.293**	9.682**	10.486**	9.212**
Adj R-squared	0.81	0.78	0.81	0.82	0.76	0.66
Durbin–Watson statistic (original)	1.2	0.96	0.88	1.15	0.65	0.91
Durbin–Watson statistic (transformed)	1.96	2.03	2.08	2.06	2.06	1.98
Rho	0.45	0.55	0.64	0.45	0.77	0.61
Observations (number of days)	505	505	505	505	505	505

Note: * Significant at the 10% level. ** Significant at the 5% level.

From the modelling results, it is quite clear that the coronavirus pandemic has had a very serious impact on footfall, though there are some differences between towns as to how this impact materialised. First, the lockdown itself had a major impact on footfall, that is, a 63% reduction in footfall for Hereford, 60% for Loughborough, 76% for Norwich, 59% for Nuneaton, 65% for Stockport and 71% for Welwyn Garden City. Second and additionally, the UK daily COVID-19 death toll seems to have had a significant negative impact on footfall for all but one town. The coefficients for Hereford and Nuneaton imply that a 10% increase in COVID-19 deaths would result in a 0.4% decrease in footfall (and vice versa), whilst for Norwich a 10% increase in deaths implies a decrease of footfall of 0.5% and finally for Loughborough and Stockport a 10% increase in deaths would lead to a decrease in footfall of 0.3%. The impact of daily deaths is not statistically significant for Welwyn Garden City.

For all towns apart from Loughborough, there was a significant negative impact on footfall after the first COVID-19 case was confirmed (this dummy variable covers the period from when the first case was detected and the start of the lockdown). Interestingly, this effect is varied, where a decrease in footfall in Norwich (decrease of 24%) and particularly Welwyn Garden City (decrease of 34%) is more pronounced compared to the other towns where the decrease ranged from 16% to 20%. For Welwyn Garden City, Figure 2 clearly indicates that footfall figures were falling rapidly before the lockdown started, indicating a town centre already in decline. Only Loughborough seems unaffected in terms of footfall, which may be explained due to the higher number of students in the town as a proportion of population than in the other towns/cities.

With regards to policy interventions related to the re-opening of Britain, some positive effects can be distinguished, in the sense that footfall has rebounded, but not as much as to fully recover as compared to pre-lockdown. The model included a variable which represents the period-of-time after the reopening of shops, and the effects are varied across the six towns/cities. Again, two groups of towns/cities can be distinguished. The first group, consisting of Hereford, Loughborough, Nuneaton and Stockport have footfall levels roughly 30% below the pre-pandemic situation. The second group of Norwich and Welwyn Garden City has footfall levels roughly 40%–45% below the pre-pandemic situation and thus had seen a very poor recovery. In speculating as to why this might be the case, Norwich is a major regional employment and retail centre and a tourist/day-trip destination, and it could be that footfall is stubbornly low due to the absence of office staff and shoppers who continued to work from home and to shop online or more locally, and tourists. Welwyn Garden City suffered footfall declines before the pandemic already, and the pandemic may have further seriously impacted on the viability of shops in the town centre. It could also be the case that both places are more seriously impacted by the pandemic, in terms of case rates and mortality.

Further, for all the towns it is quite clear that there is a Sunday effect, whereby significantly fewer people visit town centres on that day. By contrast, Saturdays seem to generate much of the footfall for Loughborough and Nuneaton (both host a local market on Saturdays) as well as Welwyn Garden City, while there is no such effect visible in Hereford or Norwich, and actually a negative effect in Stockport.

Finally, when the impact of the COVID-19 pandemic is modelled (Table 5) for all six towns/cities combined, it becomes clear that footfall declined with the arrival of COVID-19 in the UK. Since the first COVID-19 case was confirmed, footfall reduced by 17%, whilst the lockdown decreased footfall by an average of 68%. With the reopening of non-essential shops after the lockdown footfall remained 40% lower on average than during the period before COVID-19 for all the six towns and cities together. The number of daily reported COVID-19 deaths appears to have a more significant impact, where a 10% increase in COVID-19 deaths is associated with a decrease of 0.4% in footfall.

**Table 5.** Footfall in all six towns/cities combined.

Dependent variable	ln (town centre footfall)
Saturday	−0.009
Sunday	−0.448**
COVID-19 confirmed	−0.187**
Lockdown	−1.134**
Non-essential shops re-open	−0.512**
Daily deaths	−0.0004**
Constant term	11.759**
Adj R-squared	0.88
Durbin–Watson statistic (original)	0.96
Durbin–Watson statistic (transformed)	1.96
Rho	0.56
Observations	505

Note: **Significant at the 5% level.

## Discussion and managerial implications

The first point to note is that this sustained period of severe restrictions on citizen activity in the UK and in many other countries is unprecedented and follows a decade of challenging times for the High Street. In general, lockdown is bad for business, though exactly how bad varies by case (see earlier).

Second, the relaxation of the lockdown rules – that is, the re-opening of non-essential shops had a variable impact. In four towns, Hereford, Loughborough, Stockport and Nuneaton footfall has recovered more quickly than in Norwich and Welwyn Garden City. One reason could be due to differences in population profiles – for instance in the relative proportions of older people, students, white-collar workers or ethnic minorities, which then feed into their ability to work from home or shop online. Demographic factors can also relate to how people in each town are reacting to fears of becoming infected by the virus when mixing with people, and to their discomfort at being required to wear face masks in enclosed spaces (Millington et al., 2020).

Third, close examination of footfall patterns before and after COVID-19 identifies potential links between a structural decline in town centre activity and the capacity of town centres to recover in the short term from the lockdown shock – for example, in the case of Welwyn Garden City. Moreover, the results reveal local nuances. One could be the presence of a Saturday local market in the cases of Loughborough and Nuneaton, for example, which pre-pandemic were town centres with similar retail make-ups (mass market and value retail large centres). Population size may also have an impact. Thus, the larger centres of Stockport and Norwich fared much worse compared to smaller centres in the sample (i.e. considering the impact of the lockdown), perhaps because they are more dependent on longer distance commuter trips which were more likely to be substituted by people working from home as well as the service sector businesses that rely on those commuters travelling into the office. These observations suggest the importance of considering resilience as a context-specific process – namely, the critical role of the ‘five Ws’ or ‘resilience for whom, what, when, where and why’ (Meerow and Newell, 2019) is observed in the empirical evidence. Footfall patterns appear to be influenced by a combination of factors at the national level – COVID-19 measures adopted by the government nationally; at the town centre level – pre-existing trends, town centres’ characteristics and seasonal effects; as well as at the personal level – users’ demographic profile, perceptions of risk and customer experience.

Overall, the analysis suggests differential footfall patterns for the selected town centres and suggests the value of looking at footfall comparatively across multiple sites. The specific impact of contextual factors as well as the relationship of the immediate response to COVID-19 lockdowns and the pre-existing town centre performance requires further investigation. These observations also point to the need for both businesses and town centre strategists to look again at how they operate over the medium and longer term.

For businesses, there is clearly a need to consider changing patterns of consumer behaviour and how this has changed in different ways for different demographic groups. Thus, some segments have become sensitive to issues introduced by the pandemic, with some customers (e.g. older people) perceiving new risks associated with shopping to do with catching COVID-19, coming into contact with someone who is carrying COVID-19, and touching products with ‘live’ COVID-19, which consequently means that they have shifted to buying all of their products online for those or other reasons. There is also a need to consider the extent to which towns have become less attractive and the shopping experience less pleasant due to COVID-19–related adaptations to the town centre environment, for example, social distancing measures, mask-wearing, one-way pedestrian systems. In summary, businesses that survive are likely to be those which are highly adaptive, have financial resources and managerial capabilities that enable them to be responsive to the changing shopping environment, and are able to survive in a very different retail environment once COVID-19 had been to town.

Town centre managers also need to consider the role of footfall in their future regeneration strategies and the impact of new patterns of consumer behaviour after COVID-19. For instance, there could be a re-focusing of energies away from relying on large-scale events such as River Festivals or Book Fairs towards other ways of establishing value and maintaining viability. Given the new focus on social distancing, there is also a clear need for new (more flexible) approaches in how both indoor and outdoor space is managed and used. Finally, it also highlights the challenge that policy makers face in persuading citizens to adjust their behaviour – in this case it proved to be much easier to force people not to go to town centres than to encourage them to return.

## Conclusions

In conclusion, this is the first paper to study the interplay between footfall activity and resilience (as opposed to vitality) within the town centre context, and to provide detailed observations on the workings of the short-term response to shocks in practice. Specifically, it provides evidence on what has happened in terms of footfall on town centres in the immediate aftermath of coronavirus – a ‘once-in-a-lifetime’ global event which is extremely important in understanding what may happen to town centres in the longer-term and in developing appropriate management strategies (Ntounis et al., 2020). This was possible due to the application of a dynamic time series model of footfall to the six town centres, which was able to demonstrate that there was almost no auto-correlation present in the results, and hence separate the signal from the noise in the data.

Results show initial evidence of causal threads that underpin the adaptive process in town centre resilience against the coronavirus: from the pre-existing structural decline of town centres to the emergence of shock and of vulnerabilities, the immediate response and the first steps to recovery. These findings help (a) to reposition footfall as an indicator of resilience (not only vitality) and (b) to emphasise resilience as a dynamic, evolutionary and context-specific process (not merely an outcome).

This study describes only a first attempt to examine the interrelationship of footfall and resilience, focusing specifically on the immediate response to shocks, and it is not exhaustive. For example, it did not explore the relationship of footfall and other indicators of structural resilience such as vacancy rates and churn, vacant floorspace, e-presence, rent levels and tax incomes. In addition, the analysis did not examine footfall patterns at the individual business unit level, nor did it look at different product and service types and visitors’ categories. Moreover, it would be helpful to ensure more uniform coverage of each town centre in terms of the number of sensors deployed and the types of location, though this could prove challenging due to the ad hoc nature in the way that each local authority chooses to monitor footfall in their areas.

Future research could investigate further the multifarious impacts of the COVID-19 policy for different types of retailers and commercial businesses, for different locations. Furthermore, alongside footfall in terms of patterns and volumes of usage, it is necessary to also examine other characteristics of human activity such as dwell time, repeat and new visitors to better capture the ways activity relates to the multiple contributions town centres and high streets make to local communities and to the visitor experience (Hart et al., 2014). A comparative understanding of footfall alongside dwell time, for example, becomes particularly timely in the context of the COVID-19 lockdown, whereby a shift from recreational to utilitarian shopping is observed. Prior to the implementation of lockdown measures, studies on town centre customer behaviour typically treated dwell time as the primary indicator of money spent by visitors (Stocchi et al., 2016; Wrigley and Lambiri, 2014: 12). However, during the pandemic scenario, a contrasting pattern emerged which sees customers and visitors engaging in utilitarian shopping whilst minimising time spent in shops and public spaces. In this case, the volume of visitors can be more representative of the number of transactions and visitor spend compared to dwell time.

## Appendix

**Table A1.** Footfall studies in high streets, town centres and public space.

Authors	Study area	Tracking technology	Analysis method	Variables examined	Study focus
Mumford et al., 2020	155 town centres, UK	Springboard pedestrian counters	•Counts •Hourly, monthly, annual aggregation •2006 onwards; calculations with at least 2 years of data per town •K-means clustering •Principal Components Analysis (PCA) •Kruskal–Wallis test	•UK retail hierarchy •Representative year •Monthly signatures •Shopper economy, visitor economy; ‘everyday’ economy •Town types: comparison, holiday, specialty, multifunctional	•Location attractiveness •Town/city centre classification
Soundararaj et al., 2020	Pilot: Oxford Street, London, UK; Case study: 5 central London locations	•Smart Street Sensors (CDRC 2015); Wi-fi probe requests; •Android app – Clicker (manual count)	•Counts; •Sub-hourly time intervals •Clustering with sequence numbers •Calibration with ground truth •Mean absolute percentage error (MAPE)	•Installation configuration on site •Signal strength •MAPE	Unique counts from anonymised Wi-fi probe requests
Crols and Malleson, 2019	Market town Otley, Leeds Metropolitan District, UK	Wi-fi probe requests; 8 Wi-fi access points	•Counts •Hourly aggregation •August 2015–July 2017 •Agent-based modelling •generalised additive model (GAM) • Z-scores	•UK Time Use Survey (UKTUS) •Start/end time and duration of commute •Commuters vs. retired •Important activities (being at home, working, shopping, sports, having lunch, going out for food and drinks) •Peak times	•Ambient population •Daily routine simulation
Murcio et al., 2018	11 regions in Great Britain	Smart Street Sensors (CDRC 2015); 652 Wi-fi access points	•Counts •5-min time interval •‘Footfall index’ equation •Average five-minute footfall •Monthly percentage change	•Seasonal peaks •Weekday/weekend footfall volumes •Short-term and long-term footfall trends	UK footfall index
Lugomer and Longley, 2018	Great Britain shopping centres, out-of-town retail parks, high streets	Smart Street Sensors (CDRC 2015); Wi-fi probe requests; 605 Wi-fi access points	•Counts •Hourly aggregation •For 1 week •July 2015–August 2017 •Shape-based clustering methods; Dynamic Time Warping (DTW) and Euclidean distances (ED)	•Representative weekly profiles for locations •Daily footfall patterns	Temporal footfall profiles of retail microsite locations
Kim, 2018	3 city centres, Seoul, South Korea	•Wi-fi probe requests (public Wi-fi, SK Telecom, KT, and LG Uplus) •Pedestrian traffic and bank card transactions (Seoul Institute and SK Telecom)	•Counts •Hourly aggregation •Pearson’s r coefficient •Lee’s L measure •Map overlays	•Pedestrian traffic •Bank card transactions •Time slots and peak times •Age/gender groups •Region characteristics	Measuring urban vitality
Traunmueller et al., 2018	Lower Manhattan, NYC	Wi-fi probe requests; 54 Wi-fi access points	•Counts •Hourly aggregation •For 1 week •Frequencies •Journey mapping; hotspots; network graphs (k–nearest neighbour classification algorithm)	•Weekend vs. weekdays •Early morning, morning, afternoon and night •Direction of movement •Street segment usage	Mobility patterns
Lugomer et al., 2017	Sheffield and London, UK	Smart Street Sensors (CDRC 2015); Wi-fi probe requests; 2 Wi-fi access points	•Count •Shieffield: 16 × 20-min periods; London: 14 × 14-min periods •Data mining algorithms •Calibration with ground truth	•Overcounting factors •Undercounting factors	Measurement error sources
Millington et al., 2015	50 UK towns and cities	Springboard camera-based technology	•Counts •36 months, 2011–2014; •Linear regression •Percentage change •T-test for 2 signature groups	•Monthly changes •Catchment •Spend, for example, non-retail, non-food (CACI ranking) •Town/cities types: comparison, holiday, speciality, convenience/community	•Footfall as a measure of performance •Multi-functional centre profile •Activity levels in multi-functional centres
